# The bacterial community and metabolome dynamics and their interactions modulate fermentation process of whole crop corn silage prepared with or without inoculants

**DOI:** 10.1111/1751-7915.13623

**Published:** 2020-07-06

**Authors:** Dongmei Xu, Nian Wang, Marketta Rinne, Wencan Ke, Zwika G. Weinberg, Mi Da, Jie Bai, Yixin Zhang, Fuhou Li, Xusheng Guo

**Affiliations:** ^1^ State Key Laboratory of Grassland and Agro‐ecosystems School of Life Sciences Lanzhou University Lanzhou 730000 China; ^2^ Probiotics and Biological Feed Research Center Lanzhou University Lanzhou 730000 China; ^3^ Nextomics Biosciences Institute Wuhan 430000 China; ^4^ Natural Resources Institute Finland (Luke) Animale Jokioinen 31600 Finland; ^5^ Microbial Food‐Safety Research Unit Department of Food Quality and Safety The Volcani Center Agriculture Research Organization Institute for Postharvest and Food Sciences Derech HaMaccabim Road 68 POB 15159 Rishon‐LeZion 7528809 Israel

## Abstract

Multi‐omics approach was adopted to investigate the modulation of bacterial microbiota and metabolome as well as their interactions in whole crop corn ensiling systems by inoculating homofermentative *Lactobacillus plantarum* or heterofermentative *Lactobacillus buchneri*. Inoculations of the two different inoculants resulted in substantial differences in microbial community and metabolic composition as well as their dynamics in ensiled corn. Inoculants also altered the correlations of microbiota in different manners, and various keystone species were identified in corn silages with different treatments. Many metabolites with biofunctional activities like bacteriostatic, antioxidant, central nervous system inhibitory and anti‐inflammatory were found in the present silage. A constitutive difference in microbiota dynamics was found for several pathways, which were upregulated by specific taxa in middle stage of fermentation, and widespread associations between metabolites with biofunctions and the species of lactic acid bacteria dominated in silage were observed. Multiple microbial and metabolic structures and dynamics were correlated and affected the fermentation process of the corn ensiling systems. Results of the current study improve our understanding of the complicated biological process underlying silage fermentation and provide a framework to re‐evaluate silages with biofunctions, which may contribute to target‐based regulation methods to produce functional silage for animal production.

## Graphical abstract

Multi‐omics approach was adopted to investigate the modulation of bacterial microbiota and metabolome as well as their interactions in whole crop corn ensiling systems by inoculating homofermentative *Lactobacillus* plantarum or heterofermentative *Lactobacillus buchneri*. Results of the current study improve our understanding of the complicated biological process underlying silage fermentation, and provides a framework to re‐evaluate silages with biofunctions, which may contribute to target‐based reglation methods to produce functional silage for animal production.

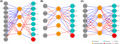

## Introduction

The anaerobic lactic acid fermentation of moist forages such as grasses, legumes, small grain cereals or corn provides an efficient way to preserve the forages (Grant and Ferraretto, 2018; Wilkinson, 2018). Corn silage, which makes up over 40% of forage fed to dairy cows (Kolver *et al*., 2001), is an important source of ensiled forage. The dairy sector is growing fast (FAO, 2013). There are over 133 million dairy cattle worldwide (Jin *et al*., 2015), which consume at least 665 million tonnes of silage per annum. About half of global ruminant meat and two third of global milk demand are estimated to be produced in developing countries by 2050, especially in China and India (Rosegrant, 2009; Gerber, 2013). Hence, nutritionally and hygienically high‐quality silage is a crucial prerequisite for developing ruminant husbandry to efficiently provide high‐quality ruminant products to the growing global population.

The biochemistry of ensiling is complex, and there are interactions among plant enzymes and the activities of microbial species (Ding *et al*., 2013). Numerous metabolites are produced during ensiling, and there are important interactions between the metabolites and the microbes as well (Guo *et al*., 2018; Xu *et al*., 2018). In addition, many metabolites are produced by lactic acid bacteria (LAB) during fermentation, such as vitamins, oligosaccharides, amino acids, aromatic compounds and fatty acids (Sun *et al*., 2012). Certain metabolites have conventionally been investigated to assess the fermentation quality of ensiled forage, but only few studies have focused on metabolites potentially affecting animal health and welfare. Therefore, improving the understanding of silage microbiome and metabolome will provide a scientific foundation for producing high‐quality silage and potentially even silages with active metabolites that may positively impact animal health and welfare.

Among the silage additives currently available, LAB strains have great potential in modulating the microbial community dynamics and end‐products during ensiling process (Kung *et al*., 2018; Liu *et al*., 2019). The most common homofermentative inoculant is *Lactobacillus plantarum,* and *Lactobacillus buchneri* 40788 is the representative of obligately heterofermentative LAB used for improve aerobic stability of silage, while it is still unclear how the homofermentative or heterofermentative LAB affect the bacterial community and metabolite dynamics in whole crop corn silage. Advanced molecular biological techniques have recently been taken into use to help understand the complex microbial communities and their succession (Pang *et al*., 2011; Keshri *et al*., 2018). However, to the best of our knowledge, no study has so far reported the population dynamics of whole crop corn silage during ensiling at species level with PacBio single molecule in conjunction with real‐time sequencing technology (SMRT). Therefore, the aim of this study was to investigate the modulation response in bacterial community and metabolome dynamics of the whole crop corn silage inoculated by a homofermentative *Lactobacillus plantarum* MTD/1 and heterofermentative *Lactobacillus buchneri* 40788.

## Results

### Fermentation characteristics of ensiled whole crop corn

The fermentation characteristics of whole crop corn silage are shown in Table 1. The pH reduction along with ensiling process was found in all treatments. The *L. buchneri* treated silage had higher pH than control or *L. plantarum* treatment except for 14 days of fermentation. The pH of *L. plantarum*‐treated samples was higher than that of the control group on 7, 30 and 45 days of ensiling. The concentration of lactic acid in all silages continued to increase over the progress of ensiling. The *L. buchneri*‐treated corn silages had lower lactic acid than control and *L. plantarum*‐treated group after ensiling for 30, 45 and 90 days respectively. Acetic acid accumulation in control and *L. plantarum*‐treated silages increased with ensiling process. The inoculant *L. buchneri* depicted the decrease of acetic acid during the first 7 days of fermentation and then constantly increase until the end of ensiling. As expected, the highest acetic acid concentration was observed in *L. buchneri*‐inoculated silage after 90 days of ensiling. The concentrations of propionic acid also indicated an increasing trend over 90 days of storage among treatments. During the first 14 days of fermentation, untreated silages showed lower propionic acid concentration than that in silages with inoculants, while the reverse result was observed on 90 days of ensiling. There was no difference on propionic acid between *L. plantarum* and *L. buchneri* treatments except for 3 days of ensiling. No butyric acid was detected in the current corn silages.

**Table 1:** Fermentation characteristics of ensiled whole crop corn.

Item	Day	Treatment			SEM	*P* value		
		C	P	B		A	T	A × T
pH	3	3.83^bA^	3.83^bA^	3.88^aA^	0.011	< 0.001	< 0.001	0.007
	7	3.75^cBC^	3.80^bA^	3.86^aA^	0.016			
	14	3.78^aAB^	3.79^aA^	3.80^aB^	0.005			
	30	3.74^bBC^	3.78^aA^	3.80^aB^	0.011			
	45	3.70^cCD^	3.73^bB^	3.77^aBC^	0.010			
	90	3.66^bD^	3.67^bC^	3.75^aC^	0.013			
LA (g kg^−1^ DM)	3	129.41^aC^	141.92^aBC^	141.41^aBC^	4.371	0.060	< 0.001	0.105
	7	147.12^aC^	138.23^aC^	135.42^aC^	3.591			
	14	161.73^aBC^	168.24^aB^	169.61^aAB^	1.789			
	30	219.17^aA^	206.84^aA^	196.96^bA^	4.476			
	45	195.56^abAB^	203.54^aA^	189.80^bA^	2.641			
	90	221.52^aA^	209.56^abA^	182.13^bA^	4.312			
AA (g kg^−1^ DM)	3	38.25^aAB^	16.51^bC^	41.14^aAB^	4.020	< 0.001	< 0.001	< 0.001
	7	37.26^aAB^	20.82^bC^	23.76^bC^	2.621			
	14	31.76^abB^	29.50^bB^	34.82^aB^	1.186			
	30	34.32^bB^	37.50^aAB^	43.33^aAB^	1.825			
	45	40.22^aAB^	38.64^aA^	34.24^bBC^	1.813			
	90	46.54^bA^	41.69^bA^	51.46^aA^	1.582			
PA (g kg^−1^ DM)	3	1.85^bD^	3.29^aD^	1.94^bD^	0.251	0.202	< 0.001	0.003
	7	2.65^bD^	3.55^aD^	3.37^abD^	0.212			
	14	5.94^bC^	7.11^aC^	7.43^aC^	0.324			
	30	9.16^aB^	8.72^aC^	8.96^aBC^	0.346			
	45	9.68^bB^	10.66^aB^	10.52^abB^	0.184			
	90	15.47^aA^	13.92^bA^	13.26^bA^	0.381			

Values with different lowercase letters (a–c) show significant differences among treatments in the same ensiling day; values with different capital letters (A–D) show significant differences among ensiling days in the same treatment (*P* < 0.05).

DM, dry matter; LA, lactic acid; AA, acetic acid; PA, propionic acid; C, control; P, *Lactobacillus plantarum*; B, *Lactobacillus buchneri*; SEM, standard error of means; A, additives; T, fermentation time (d); A × T, the interaction between additives and fermentation time.

### The inoculants altered the bacterial community composition of whole crop corn silage

**Fig. 1:**
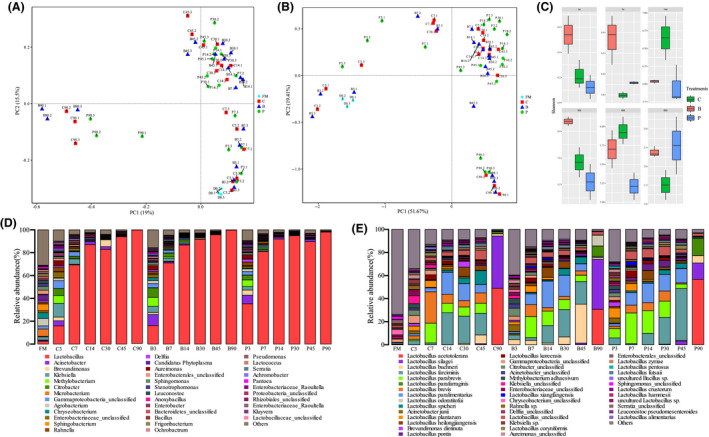
Microbial community dissimilarities and diversities of whole crop corn silage. C, Samples without inoculants; B, samples inoculated with *L. buchneri*; P, samples inoculated with *L. plantarum*; FM, fresh material. A. The community dissimilarities in different treatments and fermentation time, calculated by unweighted UniFrac distances, with coordinates calculated by principal coordinates analysis (PCoA). B. The community dissimilarities in different treatments and fermentation time, calculated by weighted UniFrac distances, with coordinates calculated by principal coordinates analysis (PCoA). C. The variations of community alpha‐diversities (Shannon index). D. Relative abundances of whole crop corn silage bacterial genus across different treatments and fermentation time. E. Relative abundances of whole crop corn silage bacterial species across different treatments and fermentation time.

Based on full‐length 16S rRNA amplicon sequencing of silage bacteria, an average of 11 948 circular consensus sequencing (CCS) sequences were obtained from each sample. The principal coordinates analysis (PCoA) based on unweighted UniFrac distances and weighted UniFrac distances was applied to identify factors that shape the differences between whole crop corn silage microbiomes (beta diversity). The results indicated a significant bacterial species succession according to fermentation time, while they were indistinguishable among silage inoculated without or with *L. plantarum* and *L. buchneri* (Fig. 1a and b). However, three distinct clusters were indentified in silages fermented for 3 to 7 days, 14 to 45 days and 90 days respectively. Especially, the microbial diversity of silages fermented for 90 days was clearly separated from that of the other fermentation times. The α‐diversity analysis revealed a decrease of the bacterial biodiversity from prolonged fermentation process in whole crop corn silage (Fig. 1c). Inoculation with *L. buchneri* increased the Shannon index at fermentation times of 3, 7 and 30 days compared with other treatments (*P* < 0.013), but there was no difference on 90 days of fermentation (*P* = 0.089). This might be due to the higher pH condition that advantages the breeding of microorganism (Ni *et al*., 2017). The *L. plantarum* decreased the Shannon index at fermentation times of 14 (*P* = 0.023) and 45 days (*P* = 0.013) compared with control. The enriched *L. parabrevis*, *L. heilongjiangensi*s and unclassified *Lactobacillus*, and *L. farciminis* and *L. paralimentarius* during 14‐ and 45‐day fermentation of *L. plantarum*‐treated corn silage, respectively, had strong competitiveness that might account for the decrease in bacteria diversity.

The composition dynamics of microbiota are shown in Fig. 1d and e. At genus level (Fig. 1d), epiphytic microflora of fresh corn was mainly comprised of undesirable bacteria for ensiling, such as *Agrobacterium* (6.42%), *Microbacterium* (6.35%), *Sphingobacterium* (5.88%), *Chryseobacterium* (5.05%) and others (31%). After 3 days of fermentation, most bacterial reads were derived from *Lactobacillus*, *Acinetobacter*, *Klebsiella*, *Methylobacterium* and *Citrobacter* in all treatments. From 7 to 90 days of fermentation, *Lactobacillus* dominated the ensiling process. The dominating species were *L. farciminis*, *L. parabrevis*, *L. brevis*, *L. parafarraginius*, *L. heilongjiangensis*, *L. acetotolerans* and *L. silagei* (Fig. 1e). Among these LAB species, *L. acetotolerans* and *L. silagei* dominated the microbial community on 90 days of fermentation. The inoculant *L. buchneri* increased the abundance of *L. buchneri* while decreased the abundance of *L. brevis* from 3 to 45 days of fermentation, and it increased the abundances of *L. parafarraginis* and *L. odoratitofui* while decreased the abundance of *L. silagei* after 90 days of fermentation. The inoculant *L. plantarum* increased the abundance of *L. parabrevis* from 3 to 30 days of fermentation and decreased the abundance of *L. farciminis* from 3 to 14 days while increased it after 45 days of ensiling. On 90 days, the inoculant *L. plantarum* resulted in an increase of *L. acetotolerans* and *L. parafarraginis* while decreased *L. silagei*.

**Fig. 2:**
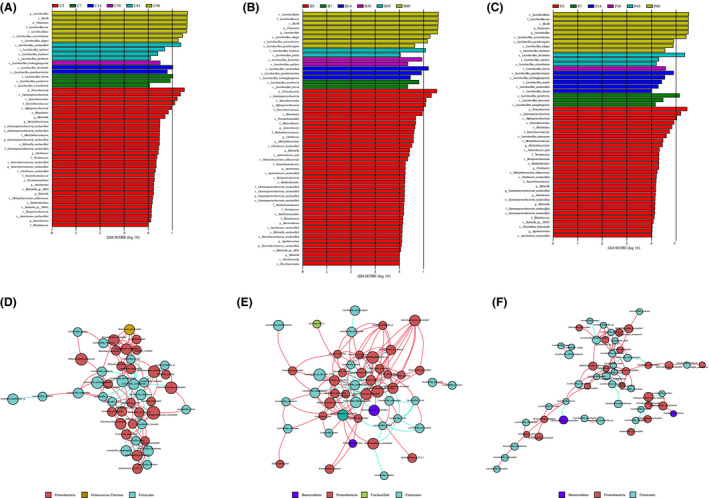
Differences of bacterial taxa in whole crop corn silage with different treatments. C, Samples without inoculants; B, samples inoculated with *L. buchneri*; P, samples inoculated with *L. plantarum*. Latent Dirichlet allocation effect size (LEfSe) analysis of whole crop corn silage bacterial biomarkers associated with inoculants for different fermentation time (A–C). Histogram of the latent Dirichlet allocation (LDA) scores computed for bacteria differentially abundant among six fermentation time. LEfSe scores can be interpreted as the degree of consistent difference in relative abundance between features in the six fermentation time of analysed microbial communities. The histogram thus identifies which bacteria taxa among all those detected as statistically and biologically differential explain the greatest differences between communities. (A) Samples without inoculants. (B) Samples inoculated with *L. buchneri*. (C) Samples inoculated with *L. plantarum*. Interaction networks of the whole crop corn silage microbiota (D–F). 16S rRNA gene‐based correlation network of the whole crop corn silage microbiota, displaying statistically significant interactions with absolute value of correlation coefficients > 0.6. Node size is scaled based on the overall abundance of each taxa in the microbiota. Edge width is proportional to the strength of association between each metabolite–phylotype pair (as measured by the correlation), red edge indicates the positive correlation, and green edge indicates the negatively correlation. (D) Samples without inoculants. (E) Samples inoculated with *L. buchneri*. (F) Samples inoculated with *L. plantarum*.

To explain the effect of differentiating fermentation process of silages treated with or without inoculants by bacterial taxa, latent Dirichlet allocation (LDA) effect size (LEfSe) analysis was conducted (Fig. 2a–c). On 3 days of fermentation, many undesirable bacteria such as *Proteobacteria*, *Gammaproteobacteria* and *Enterobacterales* were abundant in all treatments, while *Alphaproteobacteria*, *L. plantarum*, *Clostridium beijerinckii* and *Agrobacterium* were enriched in *L. plantarum‐*treated and *Alphaproteobacteria* and *Acinetobacter* were enriched in *L. buchneri*‐treated silage. In silages fermented for 7 days, *L. parabrevis* was enriched in all groups, while *L. brevis* and *L. coryniformis* were enriched in control silage, and *L. brevis* was enriched in samples treated with *L. buchneri*, and *L. koreensis* and *L. xiangfangensis* were enriched in *L. plantarum‐*treated samples. On 14 days of fermentation, *L. farciminis* and *L. paralimentarius* were enriched in control silage, while *L. heilongjiangensis* and *L. paralimentarius* were enriched in samples treated with inoculants and *L. pontis* and *L. futsaii* were enriched in *L. plantarum*‐treated silage. On 30 days of fermentation, *L. heilongjiangensis* was enriched in control silage, *L. farciminis* and *L. spicheri* were enriched in *L. buchneri*‐treated silage and *L. brevis* was enriched in *L. plantarum*‐treated silage. On 45 days of fermentation, *L. buchneri*, *L. spicheri* and *L. pantheris* were enriched in control silage; *L. buchneri* and *L. pontis* were enriched in *L. buchneri*‐inoculated silage; and *L. farciminis*, *L. spicheri* and *L. coryniformis* were enriched in *L. plantarum*‐inoculated silage. On 90 days of fermentation, the dominating species were *L. acetotolerans* and *L. silagei*, and on top of those, *L. parafarraginis* was also enriched in silage with inoculants and *L. buchneri* was also enriched in *L. plantarum*‐inoculated silage.

Microbial network was used to assess the correlation between various species and to statistically identify the bacteria species that were keystone taxa for modulating the fermentation process. The results indicated that inoculants obviously changed the correlations within microbiota (Fig. 2d–f). The putative drivers of keystone taxa in microbial communities of whole crop corn silages without or with different inoculants were defined with the combined score of high degree centrality and low betweenness centrality (Data S1). The results showed that *L. buchneri*, *L. parafarragini*s, *L. hammesii* and *Agrobacterium larrymoorei* in silage without inoculants; *L. panis* and unclassified *Enterobacteriaceae* in silage inoculated with *L. buchneri*; and *L. crustorum* and *Agrobacterium larrymoorei* in silage inoculated with *L. plantarum* can be considered as keystone taxa.

### The inoculants altered the metabolome of the whole crop corn silage

**Fig. 3:**
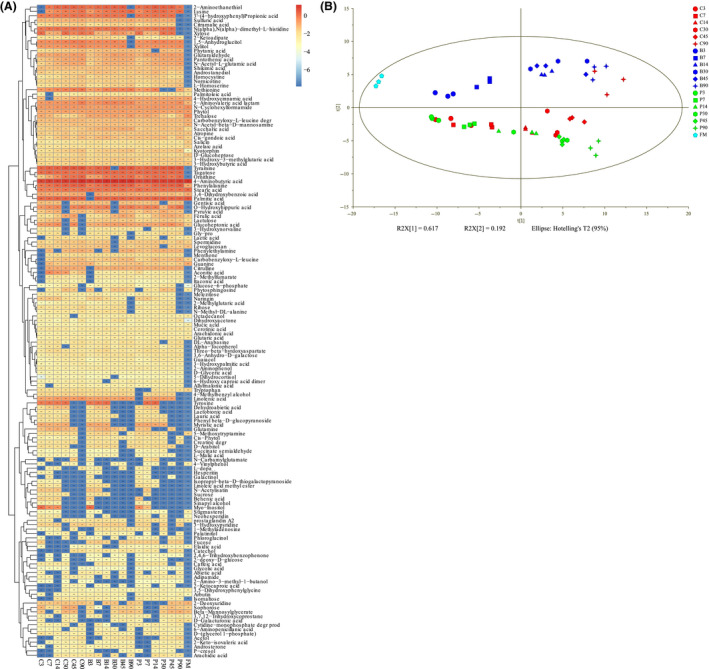
Untargeted metabolomic profile of the whole crop corn silage metabolome. C, Samples without inoculants; B, samples inoculated with *L. buchneri*; P, samples inoculated with *L. plantarum*; FM, fresh material. A. A heatmap of the relative concentrations of sum of differentially expressed metabolites. B. Principal component analysis (PCA) of metabolic profiles in whole crop corn silage inoculated without inoculation (control) or inoculated with *L. plantarum* or *L. buchneri* (*n* = 3) for different fermentation time. Input data were the total mass of the signal integration area of each sample, and the signal integration area was normalized with a method of internal standard normalization for each sample.

To evaluate the changes of metabolome of whole crop corn silage, we used the untargeted metabolomic approach. In total, 643 metabolites were identified (Data S2). The heatmap of sum of differentially expressed metabolites in various treatments and fermentation times is shown in Fig. 3a. The plot showed that many metabolites, such as amino acids, carbohydrates, organic acids, tocopherol, pantothenic acid and tyramine, were produced after fermentation. Some metabolites appeared in early period of fermentation but disappeared after certain fermentation time. Further, amino acids, sugar acids and polyhydric alcohols disappeared faster (on day 30) from inoculated silages compared to the control silage (on day 45).

The principal component analysis (PCA) of metabolome showed that the samples inoculated with *L. buchneri* were clearly separated by PC2 (second principal component), while the differences with control were not significant in samples fermented for 90 days (Fig. 3b). The control silage and samples inoculated with *L. plantarum* were separated until 45 days of fermentation. The differences of fermentation process were separated by PC1, which represented 61.7% of metabolites in ensiled forages at different fermentation times. The PC1 was mainly contributed by amino acids like leucine, 4‐aminobutyric acid, serine, alanine, phenylalanine, valine, isoleucine, proline L‐allothreonine and glycine as well as tyramine. The PC2 was mainly contributed by 4‐aminobutyric acid, 5‐aminovaleric acid lactam, methionine, stearic acid, trehalose, 2‐hydroxypyridine and lysine (Data S3). At the same time, many metabolites with biofunctional activities like bacteriostatic (naringin and 3,4‐dihydroxybenzoic acid), antioxidant (ferulic acid and catechol), central nervous system inhibitory (4‐aminobutyric acid) and anti‐inflammatory (salicin) were found in the present whole crop corn silage.

### Microbial alterations contributed to functional shifts after fermentation

**Fig. 4:**
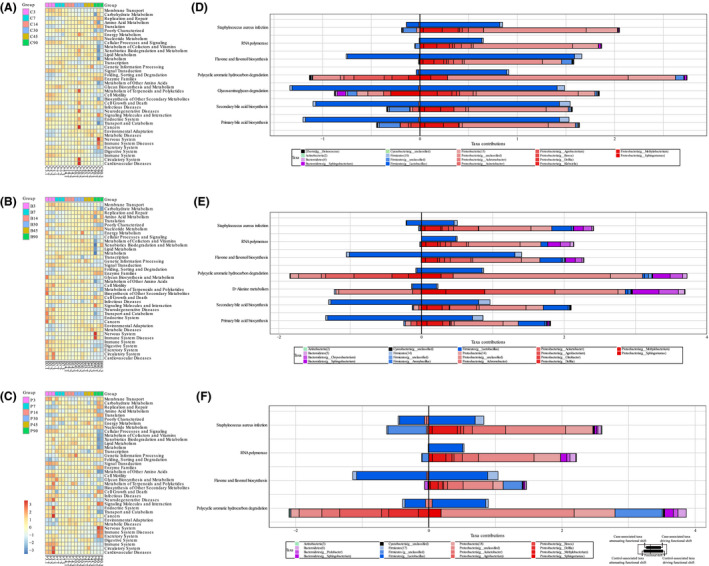
Microbial alterations contribute to functional shifts after fermentation with or without inoculants. C, Samples without inoculants; B, samples inoculated with *L. buchneri*; P, samples inoculated with *L. plantarum*. Summary of significant functional shifts predicted by the Phylogenetic Investigation of Communities by Reconstruction of Unobserved States (PICRUSt) approach (A–C). For each KEGG pathway, the second level of the predicted functional shift is shown with respect to fermentation process and treatments. A. Samples without inoculants. B. Samples inoculated with *L. buchneri*. C. Samples inoculated with *L. plantarum*. Comparing taxon‐level contribution profiles of functional shifts in fermentation process by FishTaco approach. D–F. For each KEGG pathway, the third level of the predicted functional shift is shown with respect to fermentation process and treatments. Comparison of middle stage (7–45 days of fermentation) and early stage (3 days of fermentation) of fermentation of whole crop corn silage without inoculants (D), inoculated with *L. buchneri* (E) and inoculated with *L. plantarum* (F).

In order to determine whether the observed variations in bacterial community succession contribute to community‐wide functional shifts, we used KEGG database with PICRUSt approach (Fig. 4a–c). In comparison with the control, the inoculation by *L. plantarum* and *L. buchneri* modulated the microbial communities, which resulted in marked differences in functional shift. The results indicated that the pathways closely related to silage fermentation were metabolism of carbohydrates, amino acids, energy, cofactors and vitamins and xenobiotics biodegradation and metabolism, and these pathways were upregulated in middle stage (7 to 45 days) of fermentation.

Predicted functional shifts were further examined for their association with the relative extinction or blooming of specific phylotypes. All differences between each fermentation time could not be identified statistically, so the fermentation process was divided into early period (before 7 days of fermentation, aerobic in‐silo phase in the early stage), middle period (7 to 45 days of fermentation, anaerobic fermentation) and late period (45 to 90 days of fermentation, anaerobic storage). We observed driving or attenuating functional shifts of flavones and flavonol biosynthesis, polycyclic aromatic hydrocarbon degradation, glycosaminoglycan degradation and d‐alanine metabolism (Fig. 4d–f).

Although bacterial taxa contributed to reducing functional shifts, the statistical data showed that marked upregulation of these pathways in middle period of fermentation is largely dependent on *Lactobacillus* reactions (Data S4). The pathway of glycosaminoglycan degradation and d‐alanine metabolism showed difference in control silage and samples inoculated with *L. buchneri* respectively. As for flavones and flavonol biosynthesis, differences were shown in all treatments. Compared with control silage, samples inoculated with *L. buchneri* increased the upregulation degree and the opposite result was observed in samples inoculated with *L. plantarum*. The inoculation of *L. plantarum* also decreased the upregulation of polycyclic aromatic hydrocarbon degradation pathway compared with control silage.

### Correlations between silage bacteria and well‐predicted metabolites with biofunctions

**Fig. 5:**
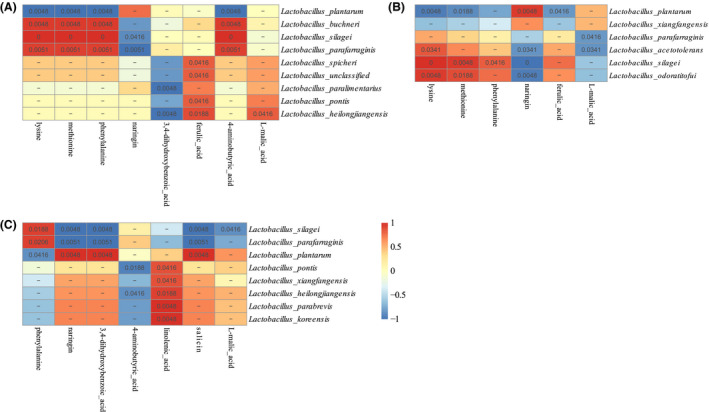
Correlation analysis of the bacteria and metabolites with biofunctional activity. Correlation visualization of significant associations between well‐predicted metabolites with biofunctions (similarity > 500, a total of 643 compounds) and bacterial phylotypes (the absolute value of correlation coefficients between metabolites and LAB species > 0.6, *P* < 0.05). The corresponding value of the heat map is the Spearman correlation coefficient *r*, which ranges between –1 and 1, *r* < 0 indicates a negative correlation (blue), *r* > 0 indicates a positive correlation (red). A. Samples without inoculants. B. Samples inoculated with *L. buchneri*. C. Samples inoculated with *L. plantarum*, *P *> 0.05.

There were widespread associations between bacteria and well‐predicted metabolites with biofunctions across the silages with or without inoculants (Data S5). The correlations between bacterial species and well‐predicted metabolites with biofunctions (lysine, methionine, phenylalanine, naringin, 3,4‐dihydroxybenzoic acid, 4‐aminobutyric acid, l‐malic acid, ferulic acid and linolenic acid) in silages with different treatments were clearly exhibited (Fig. 5a–c; the absolute value of correlation coefficients was > 0.6 between LAB and metabolites; *P* < 0.05).

Essential amino acids such as lysine, methionine and phenylalanine were detected in the present study. In control group, the *L. buchneri*, *L. silagei* and *L. parafarraginis* were positively correlated with lysine, methionine and phenylalanine, while *L. plantarum* was negatively with the three essential amino acids. In corn silage inoculated with *L. buchneri*, lysine and methionine amino acids were positively correlated with *L. silagei* and *L. odoratitofui*, lysine was also positively correlated with *L. acetotolerans*, and phenylalanine was only positively correlated with *L. silagei*. Among these essential amino acids, only phenylalanine was positively correlated with *L. silagei* and *L. parafarraginis* while negatively correlated with *L. plantarum* in corn silage treated with *L. plantarum*.

Correlations between the metabolite of naringin with bacteriostatic activity and *L. plantarum* were positive in corn silages with inoculants, while it was negatively correlated with *L. silagei* in all treatments. The naringin was also negatively correlated with *L. parafarraginis* in *L. plantarum*‐treated and control groups, but it was negatively correlated with *L. acetotolerans* and *L. odoratitofui* in the *L. buchneri*‐treated samples. Metabolite of 3,4‐dihydroxybenzoic acid was negatively correlated with *L. paralimentarius* and *L. heilongjiangensis* in control group, and it was also negatively correlated with *L. parafarraginis* and *L. silage*i in *L. plantarum*‐treated samples. However, the 3,4‐dihydroxybenzoic acid was positively correlated with *L. plantarum* in *L. plantarum*‐treated samples. Ferulic acid with antioxidant activity was also found in the present study. It was positively correlated with *L. pontis*, *L. heilongjiangensis*, *L. spicheri* and unclassified *Lactobacillus* in control group but negatively correlated with *L. plantarum* in *L. buchneri*‐treated corn silage.

*Lactobacillus parafarraginis*, *L. silagei* and *L. buchneri* were positively correlated with 4‐aminobutyric acid in control group, while *L. heilongjiangensis* and *L. pontis* were negatively correlated with it in *L. buchneri*‐inoculated samples. The linolenic acid was positively correlated with *L. parabrevis*, *L. heilongjiangensis*, *L. pontis*, *L. koreensis* and *L. xiangfangensis* in *L. plantarum*‐treated corn silage (*P *> 0.88), while no LAB was positively correlated with linolenic acid in samples inoculated with *L. buchneri* and in control group.

Salicin was positively correlated with *L. plantarum* while negatively correlated with *L. silagei* and *L. parafarraginis* in corn silage inoculated with *L. plantarum*. There was no LAB that positively correlated with salicin in samples inoculated with *L. buchneri* and in control silage. L‐malic acid was positively correlated with *L. heilongjiangensis* in control group, while it was negatively correlated with *L. parafarraginis* and *L. acetotolerans* in *L. buchneri*‐treated samples and was negatively correlated with *L. silagei* in *L. plantarum*‐treated corn silage.

### Correlations between the microbiome, metabolome and fermentation quality in whole crop corn silages

**Fig. 6:**
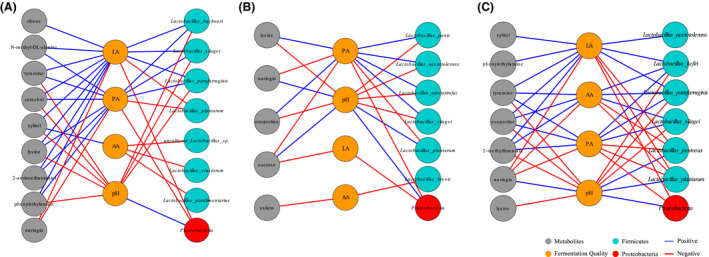
The correlation network plots for well‐predicted metabolites, bacterial taxonomic contributors and fermentation quality. Species, well‐predicted metabolites (similarity > 500) and variables of fermentation quality are presented as dots. Edge width is proportional to the strength of association (as measured by the correlation), red edge indicates negative correlation, and blue edge indicates positive correlation. The absolute value of correlation coefficients of bacterial species and fermentation quality > 0.7; the absolute value of correlation coefficients of variables of fermentation quality and well‐predicted metabolites > 0.6; *P* < 0.05. A. Samples without inoculants. B. Samples inoculated with *L. buchneri*. C. Samples inoculated with *L. plantarum*.

To further characterize the effects of bacterial species and metabolites on fermentation quality, the correlation analysis between species, well‐predicted metabolites and fermentation quality in different treatments was investigated (Fig. 6a–c). The results indicated that the quality variables of fermentation correlated positively with many species of *Lactobacillus* and negatively correlated with *Proteobacteria* (Data S6). The pH was positively correlated to *Proteobacteria* but low pH is beneficial for fermentation quality. However, *L. plantarum* in samples with inoculations and *L. pentosus* in *L. plantarum* treated silage were positively correlated with pH. The samples inoculated with *L. plantarum* increased the positive correlation between acetic acid and LAB species. The LAB strains positively correlated with fermentation quality (variables of lactic, acetic and propionic acids) were mainly tracked to *L. silagei*, *L. buchneri* and *L. parafaraginis* in control samples, to *L. silagei*, *L acetotolerans*, *L. odoratitofui* and *L. panis* in samples inoculated with *L. buchneri*, and to *L. silagei*, *L. parafaraginis*, *L. kefiri*, *L. parafaraginis* and *L acetotolerans* in *L. plantarum*‐treated samples.

The fermentation quality was closely correlated with well‐predicted metabolites such as amino acids and derivatives (phenylethylamine, lysine, N‐methyl‐dl‐alanine and oxoproline), carbohydrates (sucrose and ribose), polyphenol (catechol and naringin), alcohols (xylitol and 2‐aminoethanethiol) and 2‐methylfumarate during corn ensiling. Naringin was positively correlated with pH in inoculated samples, while it was negatively correlated with lactic and acetic acids in *L. plantarum*‐treated samples and was negatively correlated with propionic acid in all treatments. In control group, phenylethylamine, lysine, catechol and tyramine were positively correlated with lactic and propionic acid but negatively correlated with pH. Xylitol was positively correlated with lactic and acetic acids and was also negatively correlated with pH. N‐methyl‐dl‐alanine was positively correlated with propionic acid; 2‐aminoethanethiol was positively correlated with lactic and propionic acids; and ribose was positively correlated with lactic acid. In corn silage inoculated with *L. buchneri*, lysine and oxoproline were negatively correlated with pH and positively correlated with propionic acid. Salicin was negatively correlated with acetic acid. Sucrose was positively correlated with pH while negatively correlated with lactic and propionic acids. As for corn silage added with *L. plantarum*, 2‐methylfumarate, tyramine and oxoproline were negatively correlated with pH, positively correlated with lactic, acetic and propionic acids. Lysine was also negatively correlated with pH, and phenylethylamine and xylitol were positively correlated with lactic acid.

## Discussion

The rapid pH decrease in the first 3 days of ensiling is vital to inhibit undesirable microorganism and reduce nutrient loss (Ellis *et al*., 2016). The inoculation *L. plantarum* had no obvious affects on concentrations of lactic and acetic acids after 45 and 90 days of fermentation, while the *L. buchneri* decreased the concentration of lactic acid and increased the concentration of acetic acid after 30 and 90 days of ensiling. The heterofermentative LAB have been reported to improve aerobic stability by producing acetic and propionic acids. While some studies indicated that the concentrations of propionic acid were decreased by *L. buchneri* inoculation (Naiara *et al*., 2019), and the similar results were observed in whole crop corn silage on 90 days of fermentation.

To link microbial community and metabolome dynamics in corn silage to modulation by inoculants, we undertook a multi‐omics approach to characterize the changes in the microbiome and metabolome after ensiling. Interestingly, inoculants *L. buchneri* and *L. plantarum* were not the dominant species during ensiling of the corn silage, which indicated that these two applied exogenous lactic acid bacteria were not as competitive as those indigenous lactic acid bacteria species presented in the corn silage. This could be one of the possible reasons that application of *L. plantarum* is not always effective in improving the fermentation quality of corn silage (Muck and Kung, 1997), and it depends on the strain. However, the *L. plantarum* homofermentative or heterofermentative obligate *L. buchneri* modulated the microbiota and metabolome dynamics in different ways compared to the control silage although the effects of the inoculants were very dependent on the strains rather than the species. Compared with control, inoculation of *L. buchneri* increased the relative abundances of *L. paralimentarius* in silage ensiled for 7 and 14 days, and also increased *L. buchneri* in 3 to 45‐day silage and *L. parafarraginis* in 90‐day silage, while decreased the abundances of *L. brevis* in 3‐ to 45‐day silage, *L. plantarum* in 7‐ to 45‐day silage and *L. acetotolerans* in 90‐day silage. Inoculation of *L. plantarum* increased the *L. parabrevis* from 3 to 30 days of ensiling, *L. paralimentarius* in 7 and 45 days of fermentation and *L. parafarraginis* and *L. acetotolerans* in 45 and 90 days of fermentation respectively; however, it decreased the abundance of *L. silagei* in 90 days of ensiling. The dominating LAB species in whole crop corn such as *L. farciminis*, *L. parabrevis*, *L. parafarraginius*, *L. acetotolerans* and *L. silagei* were reported in our previous study (Xu *et al*., 2019). Among these species, *L. acetotolerans* is commonly, while *L. silagei* is rarely identified in corn silage. The *L. silagei* has been isolated from orchardgrass silage, and it can be cultivated on MRS agar plates (Tohno *et al*., 2013). Previous studies indicated that except for *Lactobacillus*, considerable abundances of *Weissella*, *Lactococcus*, *Pediococcus*, *Enterococcus* and *Leuconostocs* also dominate the corn silage (Brusetti *et al*., 2006; Guan *et al*., 2018). However, *Weissella*, *Pediococcus* and *Lactococcus* were not detected in the present study. The species of *Lactobacillus heilongjiangensis*, ever found in Chinese pickle (Gu *et al*., 2013), was detected for the first time in the corn silage in the present study. Furthermore, previous study reported that *L. lactic* was increased by *L. buchneri* inoculation (Parvin *et al*., 2010), while we did not detect the species in this study. The differences between studies might be due to the forage species, geographical location, period of maturity, type of fertilizer used and competition of epiphytic microflora (Mcgarvey *et al*., 2013).

The role of microbial communities in ecosystem functioning is defined (Graham *et al*., 2016; Fierer, 2017), and correlations between microorganisms are complex in the silage fermentation ecosystem. Maybe there are some species whose impact on the community is large and disproportionately large relative to its abundance (Power *et al*., 1996). This study first identified the keystone taxa with network topological properties in silage. The results indicated that inoculation altered the correlations of microflora, and the identified keystone species were totally different among the three treatment groups, which further certificated various regulations of the two types of inoculants to the microbial dynamics of the corn silage. Confusingly, the *Agrobacterium larrymoorei*, a Gram‐negative phytopathogenic bacterium, was identified as one of the keystone species both in control and *L. plantarum*‐inoculated silages, and *Enterobacteriaceae* (some species are pathogenic bacteria) was identified as keystone taxon in *L. buchneri*‐treated silage. The abundances of these two species were limited after 3 days of ensiling and disappeared after 45 days of ensiling, whether the silages have worse characteristics or not, and this needs to research further.

The end‐products during fermentation are either directly produced by microbial activity or indirectly as a result of degradation and transformation of substances present in the material. Plant enzymes in aerobic silo stage (Pahlow *et al*., 2003) and variable dynamic abundances of LAB strains resulted in dynamics of metabolome, which contributed to variations of many metabolites, particularly amino acids, in corn silage of the current research. In this study, the inoculant *L. buchneri* influenced more on metabolome than *L. plantarum*. Prediction of functional shifts over time allowed us to evaluate the impact of microbial communities on changes of metabolic pathways during ensiling. Most pathways closely related to fermentation (metabolism of carbohydrates, amino acids, energy, cofactors and vitamins, and xenobiotics biodegradation) were predicted to be upregulated in middle stage of fermentation. Although the inoculation with *L. buchneri* upregulated the flavones and flavonol biosynthesis pathway contributed by *Lactobacillus* phylotypes, a few flavonoids were detected in the present study.

In the majority of silage fermentation reactions, the substrates for lactic acid fermentation are fructose and glucose. Once the crop is cut, fructose and glucose concentrations can only increase by degradation of polysaccharides (Pahlow *et al*., 2003). Even though the fructose and glucose were not detected in this study, the glucose‐6‐phosphate is the key intermediate to understand the glucose metabolism, which increased with start of fermentation in all treatments. Thus, the glycosaminoglycan degradation pathway was significantly upregulated by *Lactobacillus* at middle stage of fermentation in non‐inoculated corn silage suggested that the polysaccharides were hydrolysed until middle stage of corn fermentation, which verified the research theories of Pahlow *et al*. (2003).

The d‐alanine metabolism pathway was upregulated by *Lactobacillus* in corn silage inoculated with *L. buchneri*. d‐alanine is an amino acid that occurs only in the peptidoglycan of bacterial cell walls (Schleifer and Kandler, 1972). Deamination can be induced by alanine to produce acetate (Li *et al*., 2018) during oxidation reaction of amino acid metabolism. However, ensiling process is under anaerobic condition, and acetic acid was produced via other pathways in the present study. Substances such as pyruvate, l‐alanine and d‐alanine involved in d‐alanine metabolism pathway have not been detected in the present study. This inconsistency might be partly due to technical difficulties in extraction and detection of some metabolites using the same oven temperature of GC‐TOF‐MS. On the other hand, the gene expression is impacted by many factors in silage fermentation like changing pH, substrates for microbiota fermentation, other metabolite regulations and interactions between microbes in the system (Pahlow *et al*., 2003; Guo *et al*., 2018; Song and Chan, 2019). Additionally, this study gives insight into to the biofunctions of silage through the associations between the metabolic output and microbial composition during ensiling. The close correlations between three essential amino acids and *L. silagei* were found in corn silage with or without inoculants. The species *L. silagei* showed higher relative abundances after 45 days of fermentation, suggesting that essential amino acid producing LAB strains can be screened from corn silage ensiled longer than 45 days. Based on the data of relative concentrations of metabolites (Data S2), the applied inoculants in the present study increased the concentration of lysine. Lysine has been reported to increase the concentration of milk protein (Weiss, 2019). Therefore, the present study suggested that inoculation of either *L. plantarum* or *L. buchneri* has potentials to improve animal performance. However, Santos *et al*. (2017) reported that *L. buchneri* 40788 had no significant effect on milk yield and protein. Thus, the practical utilize of these two inoculums on animal performance should be studied in future.

Metabolites naringin and 3,4‐dihydroxybenzoic acid with antifungal activity inhibit undesirable microorganisms to reduce nutritional losses and inhibit mycotoxin biosynthesis in silage (Haskard *et al*., 2001). These two substances showed higher relative concentrations in 3‐ and 7‐day fermented corn silage than that in silages sampled at later fermentation times. A previous study reported that *L. plantarum* with antifungal property produced phenolic or 3‐hydroxy fatty acids (Sjögren *et al*., 2003; Valan Arasu *et al*., 2013). In the current study, *L. plantarum* also positively correlated with the two metabolites irrespective of inoculants used. Thus, *L. plantarum* could be considered a species for screening inoculants with potential antifungal activity. Ferulic acid with antioxidant activity (Ggaf, 1992; Van Acker *et al*., 1996) showed different dynamics in corn silages with or without inoculants. The concentration of ferulic acid in control group decreased during 7 days of ensiling and then increased from 7 to 45 days and decreased again in 90 days of ensiling, whereas the inoculant treatments increased the ferulic acid content from 3 to 45 days while decreased in 90 days of fermentation. Ferulic acid is a hydroxycinnamic acid, and inoculants decreased the strong positive correlation of it with LAB strains. It might be because the increased ferulic acid inhibited the growth and viability (Rodríguez *et al*., 2009) of some species in inoculants treated corn silage.

4‐Aminobutyric acid, showing central nervous system inhibitory activity, blood pressure regulation and insulin secretion (Pouliot‐Mathieu *et al*., 2013; Diana *et al*., 2014) increased with the processing of fermentation in corn silage, especially in *L. buchneri*‐treated samples, which is consistent with a previous study using alfalfa silage (Guo *et al*., 2018). However, 4‐aminobutyric acid and LAB species were not correlated in *L. buchneri* treated corn silage. A number of bacteria and fungi have been reported to produce 4‐aminobutyric acid (Kono and Himeno, 2000; Lu *et al*., 2008). The most common microorganisms for 4‐aminobutyric acid production are LAB. In addition, different fermentation factors affect the 4‐aminobutyric acid production by microorganisms, and the most important ones are pH, temperature and substrate availability (Dhakal *et al*., 2012). Therefore, we speculate that many microorganisms and their dynamics as well as fermentation factors co‐affected the production of 4‐aminobutyric acid rather than abundance of certain bacterial species.

The linolenic acid may be used for the promotion of animal health and well‐being and can be produced by LAB (Salsinha *et al*., 2018). No positive correlations between linolenic acid and LAB species were detected in corn silages without inoculants and inoculated with *L. buchneri*, but there were five LAB species positively correlated with linolenic acid in *L. plantarum*‐treated corn silage. Linked to the dynamics of linolenic acid and microbial community, we found that the changes of relative abundances of *L. heilongjiangensis* and *L. pontis* were accordant with concentration of linolenic acid. Thus, the linolenic acid could be produced by *L. heilongjiangensis* and *L. pontis*. We may screen LAB strains producing linolenic acid from the two species to regulate silages with advantages on animal health and production, in future. Small amount of salicin with anti‐inflammatory activity (Albrecht *et al*., 1990) was detected during fermentation, and inoculations of *L. buchneri* and *L. plantarum* increased the relative concentration of salicin. It might be because some bacterial species utilized salicin (Wang *et al*., 2009; Cai *et al*., 2012). In addition, salicylic acid is a metabolite from salicin with antipyretic activity (Mackowiak, 2000). The relative concentration of salicylic acid was low as well. If the silage contains these two substances, it can be used to treat inflammatory conditions in periparturient or heat stressed dairy cows (Trevisi and Bertoni, 2008). We can focus on isolating or screening salicin or salicylic acid producing LAB strains as inoculants for making silages that replace antibiotic to treat inflammation in animals in further research. With many beneficial activities, malic acid is also a key intermediate in the citric acid cycle of biological tissues and has been used as a feed additive for ruminants to improve performance and efficiency (Ke *et al*., 2018). However, malic acid is metabolized to lactic acid and can be consumed during forage fermentation (Ke *et al*., 2017). In this study, L‐malic acid has not been detected in fresh corn but was present in trace amounts during ensiling, and no positive correlation with LAB species was observed in corn silages added with inoculants. It might be because the extraction and test conditions of GC‐TOF‐MS were not optimal for precisely detecting some substances, especially trace metabolites. In this regard, targeted metabolomic profiling should be conducted in further studies.

Inoculants modulated the microbial community and succession as well as dynamics of metabolites during ensiling and subsequently the fermentation quality of the corn silages. The results of multi‐omics analysis and network approaches underscore the importance of looking beyond microbiome community structure and measuring functional aspects when determining the relationship between the microbiome and silage biofunctions. In the present study, inoculations *L. buchneri* and *L. plantarum* increased the positive correlations between LAB species and fermentation quality. It is well established that selected LAB inoculants used for silage can enhance ruminant performance, *i.e*. intake, live weight gain, milk production and feed efficiency. Such effect is considered as probiotic effect of LAB inoculants (Weinberg and Muck, 1996). The microbiome and metabolome data generated in the current study give a more detailed insight into this phenomenon.

In conclusion, by means of full‐length 16S rRNA amplicon sequencing and untargeted metabolomics, we reported the complex dynamics of the whole crop corn ensiling ecosystem in terms of time‐ and inoculants‐modulated abundances of bacterial composition and multiple metabolites. The two different inoculants modified the correlations between bacterial species and the keystone species for modulating fermentation process with different manners. By following these changes, different shifts on the metabolic functions were also observed. The detected metabolites with different biofunctions indicated the potential probiotic effects of silage to animals. In addition, the correlations between metabolites and microbiome provided valuable clues to screen LAB strains with biofunctional activity, and to further develop silage inoculants that can be applied not only for improving silage fermentation quality but also could be beneficial to animal health and welfare. Therefore, the present results improved our understanding of the biological process underlying silage fermentation and may contribute to target‐based regulation methods to produce high‐quality and functional silage for animal production.

## Experimental procedures

### Ensiling and characteristics of whole crop corn silages

The whole crop corn (*Zea mays* L.; from a commercial farm of Anding county, Dingxi city, Gansu province, China) harvested at the stage of half milk‐line was chopped into a particle size of 2 cm using a forage cutter (Toyohira Agricultural Machinery, Sapporo, Japan). Three treatments were applied including a control without inoculation and inoculations using *Lactobacillus plantarum* MTD/1 (Vita Plus, Madison, MI, US) and *Lactobacillus buchneri* 40788 (Vita Plus, Madison, MI, US). The inoculants were mixed into distilled water and applied at a rate of 1 × 10^6^ cfu g^−1^ fresh matter (FM), and an equal volume of distilled water was sprayed in the fresh corn for control. The chopped whole crop corn was randomly divided into 57 batches to result in three replicates per treatment and time (3 as raw materials). Each batch was individually treated with the additives. The batches were then ensiled in vacuum‐sealed polyethylene plastic bags (30 cm × 23 cm) with about 300 g of fresh corn crop per bag. The bags were stored at ambient temperature (22–25°C) and sampled after 3, 7, 14, 30, 45 and 90 days of fermentation.

A 20 g fresh sample and silage were put in a juice exactor and squeezed with 180 ml distilled water for 30 s at a high speed, then filtered via medical gauze with four layers. The filtrate pH was measured with a glass electrode pH meter immediately. A portion of the filtrate of each sample was acidified with H_2_SO_4_ (7.14 mol l^−1^) and filtered with a 0.45‐mm dialyser. Lactic, acetic, propionic and butyric acid were analysed by high‐performance liquid chromatography (HPLC; KC‐811 column, Shodex; Shimadzu: Japan; oven temperature 50°C; flow rate 1 ml min^−1^; SPD 210 nm).

### Bacterial community composition SMRT analysis

The DNA extraction of fresh corn crop and silages was performed with a DNA isolation kit (Tiangen, DP302‐02, Tiangen, China) according to the manufacturer instructions. The PCR amplification of the full‐length 16S rRNA gene for SMRT sequencing was carried out.

Analysis of full‐length 16S rRNA amplicon sequencing data such as build of 16S rRNA library, quality control for PCR amplifications, sequences pre‐processing, species annotation and the alpha diversity was performed as described in our previous study (Xu *et al*., 2019). After the comparison with the Silva (Release132 http://www.arb‐silva.de) database (classified at a bootstrap threshold of 0.8) using the Mothur (https://mothur.org/wiki/Classify.seqs) software, the reads belonging to unclassified *Lactobacillus* were subjected to the best BLAST hit method to gain species level information (Ovaskainen *et al*., 2010; Quast *et al*., 2013) (using BLASR software). Sample ordination based on beta diversity was examined by means of PCoA with phylogeny‐based (UniFrac) unweighted and weighted distances (using QIIME). LEfSe method was used to determine the genes most likely to explain differences between treatments by coupling standard tests for statistical significance with additional tests encoding biological consistency and effect relevance (Segata *et al*., 2011). Microbial networks were used to statistically identify keystone taxa, and the combined score of high mean degree and low betweenness centrality was used as a threshold for defining keystone taxa in microbial communities (Berry and Widder, 2014). Phylogenetic Investigation of Communities by Reconstruction of Unobserved States (PICRUSt) was used to predict the metagenome in terms of Kegg Orthology (KO) terms for each 16S rRNA sample (Langille *et al*., 2013). Microbiome functional shifts and phylotype‐level contributions to functional shifts were obtained using the FishTaco framework (Manor and Borenstein, 2017).

### Metabolomics using GC‐TOF‐MS

The method of extraction was described in our previous study (Xu *et al*., 2019). The quality control (QC) samples consisted of partial extract (75 μl) from each sample. Samples were analysed by an Agilent 7890 gas chromatograph system coupled with a Pegasus HT time‐of‐flight mass spectrometer (GC‐TOF‐MS). The system used a DB‐5MS capillary column coated with 5% diphenyl and cross‐linked with 95% dimethylpolysiloxane (30 m × 250 μm inner diameter, 0.25 μm film thickness; J&W Scientific, Folsom, CA, USA). Samples (1 µl) were injected in split mode (split ratio 20:1), with helium used as the carrier gas at a flow rate of 1.0 ml min^−1^. The oven temperature ramp was as follows: initial temperature was 50°C for 1 min, raised to 310°C at a rate of 10°C min^−1^ and finally kept at 310°C for 8 min. The injection, transfer line and ion source temperatures were 280, 280 and 250°C respectively. The energy was −70 eV in electron impact mode. The mass spectrometry data were acquired in full‐scan mode with an m z^−1^ range of 50–500 at a rate of 12.5 spectra per second, after a solvent delay of 6.17 min.

Raw peak exaction, data baseline filtration and calibration of the baseline, as well as peak alignment, deconvolution analysis, peak identification and integration of the peak area were performed with Chroma TOF 4.3X software of LECO Corporation and the LECO‐Fiehn Rtx5 database (Kind *et al*., 2009). Both the mass spectrum match and retention index match were considered in metabolites identification. Peaks with poor repeatability (< 50% of QC samples or RSD > 30%) in QC samples were removed (Dunn *et al*., 2011). The NIST (http://www.nist.gov/index.html) and KEGG (http://www.genome.jp/kegg/) commercial databases were used to identify metabolites.

For statistical analysis, missing values were assumed to be below the level of detection. However, metabolites were detected in all samples from one or more groups but not in samples from other groups, which were assumed to be near the lower limit of detection in the groups in which they were not detected (Theriot *et al*., 2014). We further normalized the entire set of 57 samples using SIMCA software (version 14, Umetrics AB, Umea, Sweden). PCA models were tested for all samples. Welch’s two‐sample *t*‐test was used to identify biochemicals that differed significantly between experimental groups. We defined significantly different compounds between treatment groups by the criteria of VIP > 1 (first principal component of orthogonal projections to latent structure discriminant analysis (OPLS‐DA)) and *P*‐value < 0.05.

### Microbiome, metabolome and fermentation quality correlation analysis

We computed the Spearman’s rank correlation coefficients for bacterial species and all identified metabolites with biofunctions (a total of 643 compounds). We performed heatmap construction as a graph and annotated species by the phylum (*Proteobacteria* and *Firmicutes*) and well‐predicted metabolites (similarity > 500) with biofunctions. To further characterize the effects of bacterial species and metabolites on fermentation quality, the Spearman’s correlation analysis between bacterial species, identified metabolites and fermentation quality in different treatments was computed and network plots were performed by Cytoscape (v 3.6.1). In order to show the plots clearly, we screened only part of the data. Briefly, the well‐predicted metabolites were selected with similarity > 500, and then, the absolute value of correlation coefficients of bacterial species and fermentation quality was > 0.7; the absolute value of correlation coefficients of variables of fermentation quality and well‐predicted metabolites was > 0.6; *P* < 0.05.

## Conflict of interest

None declared.

## Supporting information


**Data S1.** Betweenness centrality and degree centrality of the microbial network analysis for corn silage without inoculants (Fig. 2d).

**Data S2.** Relative concentration of detected substances in corn before and after ensiling for 3, 7, 14, 30, 45 and 90 days. Identified and unidentified substances (Analyte ** and unknown).

**Data S3.** The contribution of 634 metabolites to the first principal component (PC1) and standard error (Fig. 3b).

**Data S4.** Wilcoxon test statistics for significant functional shifts predicted by FishTaco approach in different tretments (Fig. 4d–f).

**Data S5.** Spearman correlation coefficients of that between microbiota and metabolites with biofunctions in corn silage inoculated with *L. plantarum* (Fig. 5c). The absolute Spearman.

**Data S6.** Spearman correlation coefficients of that between microbiota, well‐predicted metabolites and variables of fermentation quality in corn silage inoculated with *L. plantarum*.

## Data Availability

Raw sequencing files and associated metadata have been deposited in NCBI’s Sequence Read Archive (accession PRJNA565131), https://www.ncbi.nlm.nih.gov/sra.

## References

[mbt213623-bib-0001] Albrecht, M. , Nahrstedt, A. , Luepke, N.P. , Theisen, N.L. , and Baron, G. (1990) Anti‐inflammatory activity of flavonol glycosides and salicin derivatives from the leaves of Populus tremuloides. Planta Med 56: 660.

[mbt213623-bib-0002] Berry, D. , and Widder, S. (2014) Deciphering microbial interactions and detecting keystone species with co‐occurrence networks. Front Microbiol 5: 1–15. 24904535 10.3389/fmicb.2014.00219 PMC4033041

[mbt213623-bib-0003] Brusetti, L. , Borin, S. , Mora, D. , Rizzi, A. , Raddadi, N. , Sorlini, C. , and Daffonchio, D. (2006) Usefulness of length heterogeneity‐PCR for monitoring lactic acid bacteria succession during maize ensiling. FEMS Microbiol Ecol 56: 154–164. 16542413 10.1111/j.1574-6941.2005.00059.x

[mbt213623-bib-0004] Cai, Y. , Pang, H. , Kitahara, M. , and Ohkuma, M. (2012) Lactobacillus nasuensis sp. nov., a lactic acid bacterium isolated from silage, and emended description of the genus Lactobacillus. Int J Syst Evol Microbiol 65: 1140–1144. 10.1099/ijs.0.031781-0 21724957

[mbt213623-bib-0005] Dhakal, R. , Bajpai, V.K. , and Baek, K. (2012) Production of GABA (γ‐aminobutyric acid) by microorganisms: a review. Braz J Microbiol 43: 1230–1241. 24031948 10.1590/S1517-83822012000400001 PMC3769009

[mbt213623-bib-0006] Diana, M. , Quílez, J. , and Rafecas, M. (2014) Gamma‐aminobutyric acid as a bioactive compound in foods: a review. J Funct Foods 10: 407–420.

[mbt213623-bib-0007] Ding, W.R. , Long, R.J. , and Guo, X.S. (2013) Effects of plant enzyme inactivation or sterilization on lipolysis and proteolysis in alfalfa silage. J Dairy Sci 96: 2536–2543. 23415523 10.3168/jds.2012-6438

[mbt213623-bib-0008] Dunn, W.B. , Broadhurst, D. , Begley, P. , Zelena, E. , Francis‐mcintyre, S. , Anderson, N. , *et al*. (2011) Procedures for large‐scale metabolic profiling of serum and plasma using gas chromatography and liquid chromatography coupled to mass spectrometry. Nat Protoc 6: 1060–1083. 21720319 10.1038/nprot.2011.335

[mbt213623-bib-0010] Ellis, J.L. , Hindrichsen, I.K. , Klop, G. , Kinley, R.D. , Milora, N. , Bannink, A. , and Dijkstra, J. (2016) Effects of lactic acid bacteria silage inoculation on methane emission and productivity of Holstein Friesian dairy cattle. J Dairy Sci. 99: 7159–7174. 27372595 10.3168/jds.2015-10754

[mbt213623-bib-0011] FAO (2013) The Global Dairy Sector: Facts.

[mbt213623-bib-0012] Fierer, N. (2017) Embracing the unknown: disentangling the complexities of the soil microbiome. Nat Rev Microbiol 15: 579. 28824177 10.1038/nrmicro.2017.87

[mbt213623-bib-0013] Gerber, P.J. (2013) Tackling Climate Change through Livestock – A Global Assessment of Emissions and Mitigation Opportunities. Rome, Italy: Food and Agriculture Organization of the United Nations.

[mbt213623-bib-0014] Ggaf, E. (1992) Antioxidant potential of ferulic acid. Free Radical Bio Med 13: 435–448. 1398220 10.1016/0891-5849(92)90184-i

[mbt213623-bib-0015] Graham, E.B. , Je, K. , Schindlbacher, A. , Siciliano, S. , Hc, G. , Dl, J. , *et al*. (2016) Microbes as engines of ecosystem function: when does community structure enhance predictions of ecosystem processes ? Front Microbiol 7: 214. 26941732 10.3389/fmicb.2016.00214 PMC4764795

[mbt213623-bib-0016] Grant, R.J. , and Ferraretto, L.F. (2018) Silage review: Silage feeding management : silage characteristics and dairy cow feeding behavior. J Dairy Sci 101: 4111–4121. 29685280 10.3168/jds.2017-13729

[mbt213623-bib-0017] Gu, C.T. , Li, C.Y. , Yang, L.J. , and Huo, G.C. (2013) *Lactobacillus heilongjiangensis* sp. nov., isolated from Chinese pickle. Int J Syst Evol Microbiol 63: 4094–4099. 23728376 10.1099/ijs.0.053355-0

[mbt213623-bib-0018] Guan, H. , Yan, Y. , Li, X. , Li, X. , Shuai, Y. , Feng, G. , *et al*. (2018) Microbial communities and natural fermentation of corn silages prepared with farm bunker‐silo in Southwest China. Biores Technol 265: 282–290. 10.1016/j.biortech.2018.06.018 29908496

[mbt213623-bib-0019] Guo, X.S. , Ke, W.C. , Ding, W.R. , Ding, L.M. , Xu, D.M. , Wang, W.W. , *et al*. (2018) Profiling of metabolome and bacterial community dynamics in ensiled *Medicago sativa* inoculated without or with *Lactobacillus plantarum* or *Lactobacillus buchneri* . Sci Rep 8: 1–10. 29321642 10.1038/s41598-017-18348-0 PMC5762819

[mbt213623-bib-0021] Haskard, C.A. , El‐Nezami, H.S. , Kankaanpaa, P. , Salminen, S. , and Ahokas, J. (2001) Surface binding of aflatoxin B1 by lactic acid bacteria. Appl Environ Microbiol 67: 3086–3091. 11425726 10.1128/AEM.67.7.3086-3091.2001 PMC92985

[mbt213623-bib-0022] Jin, L. , Duniere, L. , Lynch, J.P. , Mcallister, T.A. , Baah, J. , and Wang, Y. (2015) Impact of ferulic acid esterase producing Lactobacilli and fibrolytic enzymes on conservation characteristics, aerobic stability and fiber degradability of barley silage. Anim Feed Sci Technol 207: 62–74.

[mbt213623-bib-0023] Ke, W.C. , Ding, W.R. , Xu, D.M. , Ding, L.M. , Zhang, P. , Li, F.D. , and Guo, X.S. (2017) Effects of addition of malic or citric acids on fermentation quality and chemical characteristics of alfalfa silage. J Dairy Sci 100: 8958–8966. 28918135 10.3168/jds.2017-12875

[mbt213623-bib-0024] Ke, W.C. , Ding, W.R. , Xu, D.M. , Ding, L.M. , Zhang, P. , Li, F.D. , and Guo, X.S. (2018) Influences of malic acid isomers and their application levels on fermentation quality and biochemical characteristics of alfalfa silage. Anim Feed Sci Technol 245: 1–9.

[mbt213623-bib-0025] Keshri, J. , Chen, Y. , Pinto, R. , Kroupitski, Y. , Weinberg, Z.G. , and Saldinger, S.S. (2018) Microbiome dynamics during ensiling of corn with and without *Lactobacillus plantarum* inoculant. Appl Microbiol Biotechnol 102: 4025–4037. 29536147 10.1007/s00253-018-8903-y

[mbt213623-bib-0026] Kind, T. , Wohlgemuth, G. , Lee, D.Y. , Lu, Y. , Palazoglu, M. , Shahbaz, S. , and Fiehn, O. (2009) FiehnLib: mass spectral and retention index libraries for metabolomics based on quadrupole and time‐of‐flight gas chromatography/mass spectrometry. Anal Chem 81: 10038–10048. 19928838 10.1021/ac9019522 PMC2805091

[mbt213623-bib-0027] Kolver, E.S. , Roche, J.R. , DMiller, D. , and Densley, R. (2001) Maize silage for dairy cows. In Proceedings of the conference‐New Zealand Grassland Association, pp. 195–202.

[mbt213623-bib-0028] Kono, I. , and Himeno, K. (2000) Changes in γ‐aminobutyric acid content during beni‐koji making. Biosci Biotechnol Biochem 64: 617–619. 10803966 10.1271/bbb.64.617

[mbt213623-bib-0029] Kung, L. Jr , Shaver, R.D. , Grant, R.J. , and Schmidt, R.J. (2018) Silage review: Interpretation of chemical, microbial, and organoleptic components of silages. J Dairy Sci 101: 4020–4033. 29685275 10.3168/jds.2017-13909

[mbt213623-bib-0030] Langille, M.G.I. , Zaneveld, J. , Caporaso, J.G. , Mcdonald, D. , Knights, D. , Reyes, J.A. , *et al*. (2013) Predictive functional profiling of microbial communities using 16S rRNA marker gene sequences. Nat Biotechnol 31: 814. 23975157 10.1038/nbt.2676 PMC3819121

[mbt213623-bib-0031] Li, S.Y. , Ng, I.S. , Chen, P.T. , Chiang, C.J. , and Chao, Y.P. (2018) Biotechnology of protein waste for production of sustainable fuels and chemicals. Biotechnol Biofuels 11: 256. 30250508 10.1186/s13068-018-1234-5 PMC6146663

[mbt213623-bib-0032] Liu, B. , Huan, H. , Gu, H. , Xu, N. , Shen, Q. , and Ding, C. (2019) Dynamics of a microbial community during ensiling and upon aerobic exposure in lactic acid bacteria inoculation‐treated and untreated barley silages. Bioresour Technol 273: 212–219. 30447622 10.1016/j.biortech.2018.10.041

[mbt213623-bib-0033] Lu, X. , Chen, Z. , Gu, Z. , and Han, Y. (2008) Isolation of γ ‐aminobutyric acid‐producing bacteria and optimization of fermentative medium. Biochem Eng J 41: 48–52.

[mbt213623-bib-0034] Mackowiak, P.A. (2000) Brief history of antipyretic therapy. Clin Infecti Dis 31: 154–156. 10.1086/317510 11113017

[mbt213623-bib-0035] Manor, O. , and Borenstein, E. (2017) Systematic characterization and analysis of the taxonomic drivers of functional shifts in the human microbiome. Cell Host Microbe 21: 1–14. 28111203 10.1016/j.chom.2016.12.014 PMC5316541

[mbt213623-bib-0036] Mcgarvey, J.A. , Franco, R.B. , Palumbo, J.D. , Hnasko, R. , Stanker, L. , and Mitloehner, F.M. (2013) Bacterial population dynamics during the ensiling of *Medicago sativa* (alfalfa) and subsequent exposure to air. J Appl Microbiol 114: 1661–1670. 23521112 10.1111/jam.12179

[mbt213623-bib-0037] Muck, R.E. , and Kung, L. Jr (1997) Effects of silage additives on ensiling. In Silage Field to Feedbunk, NRAES‐99. Ithaca, NY: Northeast Regional Agric. Engng. Service, pp. 187–199.

[mbt213623-bib-0038] Naiara, C. , Nascimento, C.F. , Campos, V.M.A. , Alves, M.A.P. , Resende, F.D. , Daniel, J.L.P. , and Siqueira, G.R. (2019) Influence of storage length and inoculation with *Lactobacillus buchneri* on the fermentation, aerobic stability, and ruminal degradability of high‐moisture corn and rehydrated corn grain silage. Anim Feed Sci Technol 251: 124–133.

[mbt213623-bib-0039] Ni, K. , Wang, F. , Zhu, B. , Yang, J. , Zhou, G. , Pan, Y. , and Zhong, J. (2017) Effects of lactic acid bacteria and molasses additives on the microbial community and fermentation quality of soybean silage. Bioresour Technol 238: 706–715. 28501002 10.1016/j.biortech.2017.04.055

[mbt213623-bib-0040] Ovaskainen, O. , Nokso‐koivisto, J. , Hottola, J. , and Rajala, T. (2010) Identifying wood‐inhabiting fungi with 454 sequencing – what is the probability that BLAST gives the correct species? Fungal Ecol 3: 274–283.

[mbt213623-bib-0041] Pahlow, G. , Muck, R.E. , Driehuis, F. , and Oude Elferink, S.J.W.H. (2003) Silage Science and Technology. Agronomy Monograph, no. 42. Madison, WI: American Society of Agronomy, Crop Science Society of America, Soil Science Society of America.

[mbt213623-bib-0042] Pang, H.L. , Qin, G.Y. , Tan, Z.F. , Li, Z.W. , Wang, Y. , and Cai, Y. (2011) Natural populations of lactic acid bacteria associated with silage fermentation as determined by phenotype, 16S ribosomal RNA and recA gene analysis. Syst Appl Microbiol 34: 235–241. 21282025 10.1016/j.syapm.2010.10.003

[mbt213623-bib-0043] Parvin, S. , Wang, C. , Li, Y. , and Nishino, N. (2010) Effects of inoculation with lactic acid bacteria on the bacterial communities of Italian ryegrass, whole crop maize, guinea grass and rhodes grass silages. Anim Feed Sci Technol 160: 160–166.

[mbt213623-bib-0044] Pouliot‐Mathieu, K. , Gardner‐Fortier, C. , Lemieux, S. , St‐Gelais, D. , Champagne, C.P. , and Vuillemard, J.‐C. (2013) Effect of cheese containing gamma‐aminobutyric acid‐producing lactic acid bacteria on blood pressure in men. PharmaNutrition 1: 141–148.

[mbt213623-bib-0045] Power, M.E. , Tilman, D. , Estes, J.A. , Menge, B.A. , Bond, W.J. , Mills, L.S. , *et al*. (1996) Challenges in the quest for keystones: identifying keystone species is difficult—but essential to understanding how loss of species will affect ecosystems. Bioscience 46: 609–620.

[mbt213623-bib-0046] Quast, C. , Pruesse, E. , Yilmaz, P. , Gerken, J. , Schweer, T. , Yarza, P. , *et al*. (2013) The SILVA ribosomal RNA gene database project: Improved data processing and web‐based tools. Nucleic Acids Res 41: 590–596. 10.1093/nar/gks1219 PMC3531112 23193283

[mbt213623-bib-0047] Rodríguez, H. , Antonio, J. , María, J. , De, B. , López, F. , Felipe, D. , *et al*. (2009) Food phenolics and lactic acid bacteria. Int J Food Microbiol 132: 79–90. 19419788 10.1016/j.ijfoodmicro.2009.03.025

[mbt213623-bib-0048] Rosegrant, M.W. (2009) International Assessment of Agricultural Knowledge, Science and Technology for Development (IAASTD). Global Rreport. Washington, DC: Island Press.

[mbt213623-bib-0049] Salsinha, A.S. , Pimentel, L.L. , Fontes, A.L. , and Gomes, A.M. (2018) Microbial production of conjugated linoleic acid and conjugated linolenic acid relies on a multienzymatic system. Microbiol Mol Biol Rev 82: e00019‐18. 30158254 10.1128/MMBR.00019-18 PMC6298612

[mbt213623-bib-0050] Santos, W.P. , Vila, C.L.S. , Pereira, M.N. , Schwan, R.F. , Lopes, N.M. , and Pinto, J.C. (2017) Effect of the inoculation of sugarcane silage with *Lactobacillus hilgardii* and *Lactobacillus buchneri* on feeding behavior and milk yield of dairy cows1. J Anim Sci 95: 4613–4622. 29108036 10.2527/jas2017.1526

[mbt213623-bib-0051] Schleifer, K.H. , and Kandler, O. (1972) Peptidoglycan types of bacterial cell walls and their taxonomic implications. Bacteriol Rev 36: 407–477. 4568761 10.1128/br.36.4.407-477.1972 PMC408328

[mbt213623-bib-0052] Segata, N. , Izard, J. , Waldron, L. , Gevers, D. , Miropolsky, L. , Garrett, W.S. , and Huttenhower, C. (2011) Metagenomic biomarker discovery and explanation. Genome Biol 12: R60. 21702898 10.1186/gb-2011-12-6-r60 PMC3218848

[mbt213623-bib-0053] Sjögren, J. , Magnusson, J. , Broberg, A. , Schnürer, J. , and Kenne, L. (2003) Antifungal 3‐hydroxy fatty acids from *Lactobacillus plantarum* MiLAB 14. Appl Environ Microbiol 69: 7554–7557. 14660414 10.1128/AEM.69.12.7554-7557.2003 PMC309954

[mbt213623-bib-0054] Song, M. , and Chan, A.T. (2019) Environmental factors, gut microbiota, and colorectal cancer prevention. Clin Gastroenterol Hepatol 17: 275–289. 30031175 10.1016/j.cgh.2018.07.012 PMC6314893

[mbt213623-bib-0055] Sun, M.‐C. , Li, A.‐L. , Huo, G.‐C. , and Meng, X.‐C. (2012) Progress on the metabolomics of lactic acid bacteria. Microbiology/Weishengwuxue Tongbao 39: 1499–1505.

[mbt213623-bib-0056] Theriot, C.M. , Koenigsknecht, M.J. Jr , Hatton, P.E.C. , Nelson, G.E. , Li, A.M. , Huffnagle, B. , *et al*. (2014) Antibiotic‐induced shifts in the mouse gut microbiome and metabolome increase susceptibility to Clostridium difficile infection. Nat Commun 5: 3114. 24445449 10.1038/ncomms4114 PMC3950275

[mbt213623-bib-0057] Tohno, M. , Kitahara, M. , Irisawa, T. , Masuda, T. , and Tajima, K. (2013) *Lactobacillus silagei sp*. nov. isolated from orchardgrass silage. Int J Syst Evol Microbiol 63: 12. 10.1099/ijs.0.053124-0 23919960

[mbt213623-bib-0058] Trevisi, E. , and Bertoni, G. (2008) Attenuation with acetylsalicylate treatments of inflammatory conditions in periparturient dairy cows. Aspirin Health Res Prog, 22–37.

[mbt213623-bib-0059] Valan Arasu, M. , Jung, M.W. , Ilavenil, S. , Jane, M. , Kim, D.H. , Lee, K.D. , *et al*. (2013) Isolation and characterization of antifungal compound from *Lactobacillus plantarum* KCC‐10 from forage silage with potential beneficial properties. J Appl Microbiol 115: 1172–1185. 23910250 10.1111/jam.12319

[mbt213623-bib-0060] Van Acker, S.A. , Tromp, M.N. , Griffioen, D.H. , Van Bennekom, W.P. , Van Der Vijgh, W.J. , and Bast, A. (1996) Structural aspects of antioxidant activity of flavonoids. Free Radical Bio Med 20: 331–342. 8720903 10.1016/0891-5849(95)02047-0

[mbt213623-bib-0061] Wang, L.T. , Kuo, H.P. , Wu, Y.C. , Tai, C.J. , and Lee, F.L. (2009) Lactobacillus taiwanensis sp. nov., isolated from silage. Int J Syst Evol Microbiol 59: 2064–2068. 19605711 10.1099/ijs.0.006783-0

[mbt213623-bib-0062] Weinberg, Z.G. , and Muck, R.E. (1996) New trends and opportunities in the development and use of inoculants for silage. FEMS Microbiol Rev 19: 53–68.

[mbt213623-bib-0063] Weiss, W.P. (2019) Effects of feeding diets composed of corn silage and a corn milling product with and without supplemental lysine and methionine to dairy cows. J Dairy Sci 102: 2075–2084. 30612798 10.3168/jds.2018-15535

[mbt213623-bib-0064] Wilkinson, J.M. (2018) Highlights of progress in silage conservation and future perspectives. Grass Forage Sci 73: 40–52.

[mbt213623-bib-0065] Xu, Z. , Zhang, S. , Zhang, R. , Li, S. , and Kong, J. (2018) The changes of dominant lactic acid bacteria and their metabolites during corn stover ensiling. J Appl Microbiol. 10.1111/jam.13914 29762882

[mbt213623-bib-0066] Xu, D. , Ding, W. , Ke, W. , Li, F. , Zhang, P. , and Guo, X. (2019) Modulation of metabolome and bacterial community in whole crop corn silage by inoculating homofermentative *Lactobacillus plantarum* and heterofermentative *Lactobacillus buchneri* . Front Microbiol 9: 1–14. 10.3389/fmicb.2018.03299 PMC6352740 30728817

